# An experiment of health services and additional microcredit in 128 villages of Bangladesh

**DOI:** 10.1186/s41043-022-00292-z

**Published:** 2022-05-02

**Authors:** Stan Becker, Ruhul Amin, Nirali Chakraborty, Linnea Zimmerman

**Affiliations:** 1 Department of Population, Family and Reproductive Health, Johns Hopkins University, Baltimore, MD USA; 2 Director of Research and Technical Assistance, Metrics for Management, Baltimore, MD USA

**Keywords:** Microcredit, Experimental study, Health services intervention, Bangladesh, Asia, Randomized controlled trial

## Abstract

**Background:**

Studies in the literature have found mixed results on the effect of microcredit on health outcomes. Of the five previous experimental studies that included microcredit and a health intervention, three reported no significant changes in health status or behaviors. The purpose of this study was to test for marginal and interactive effects of increased microcredit and provision of basic health services.

**Methods:**

This study had a 4-celled experimental design in 128 villages in rural Bangladesh. For villages in one cell, an additional microcredit worker was assigned. For those in a second cell, a health assistant visited households each month, provided simple medicines and announced a satellite clinic held monthly in each village. For a third cell, both interventions were combined, and villages in a fourth cell served as control. A baseline survey was completed and a follow-up survey was done three years later. Outcome measures were food security, contraceptive use, having a trained birth attendant at last birth, and measles immunization.

**Results:**

Comparison of follow-up with baseline levels of the four outcome measures (for 3787 households (96% completeness) and 3687 women (94% completeness)) showed significant improvement in food security in all study arms and a significant increase in trained birth attendant at last birth in the health services villages. Due to confusion within Grameen Bank about which workers would provide the additional microcredit work, that intervention was poorly implemented so in multivariate analyses, the data for that intervention arm were grouped with data from the control arm. Logistic regression with values of the outcomes at follow-up as dependent variable and study arm and women’s schooling as covariates showed no significant effects of either separate or grouped study arms.

**Conclusion:**

Two of the three health behaviors showed no significant changes over time but having a trained birth attendant at last delivery did increase significantly in the health services arm. Therefore, community health education can sometimes be effective in promoting healthy behaviors.

***Trial registration*:**

This was a field trial rather than a clinical trial, so trial registration was unnecessary.

## Background

Microcredit—initially introduced by Professor Mohammed Yunus in a tiny village of Bangladesh in 1976 [34]—is now available in many countries with over 1000 relatively large microfinance/microcredit institutions (MFIs) and 140 million clients receiving grants as of 2019 [7]. While the positive effect of microcredit on women and households seemed to be clear from the early Bangladesh experience, six recent randomized controlled trials (RCTs) of microcredit have not found significant effects on either income, consumption or poverty [2–5, 9, 32]. Earlier cross-sectional studies suffered from selection biases that could explain why they found significant results while the RCTs generally have not. Pitt et al. [26] outlined the three types of biases in the cross-sectional studies: (1) choice-based sampling, (2) self-selection into microcredit, and (3) non-random program placement. In some cases econometric methods can be used to adjust for one or more of these biases. With RCTs, these biases are either eliminated (with intent-to-treat analyses) or minimized. Earlier studies with econometric methods found that women’s participation in microcredit led to increases in household income, production and expenditures [26] (Khandker and Khan 1998). However, analytic methods used in the Pitt et al. study have been debated and a reanalysis with different assumptions did not replicate the significant results of microcredit on poverty reduction [27].

Regarding the potential effects of microcredit on health, the positive health impact of microcredit has been postulated as an important outcome for users and their families [17, 18]. Some programs use microcredit clients’ meetings to teach good health practices, family planning, and other aspects of reproductive health. Others use microcredit services to promote health insurance [8]. A theoretical underpinning linking microcredit and health has been given by Mohindra and Haddad [23]. They posit four pathways linking the two: economic, social, psychological, and political. The economic pathway is straightforward because of the “close link between wealth and health,” the social pathway involves social networks, changing norms and attitudes and increasing women’s social engagement and participation; and the psychological pathway involves the concept of self-efficacy. Finally, the political pathway gives women “voice” to influence public policies and interventions that impact women’s health.

With regard to studies of health status and/or behaviors *vis-à-vis* microcredit, several types of studies in the literature need to be distinguished. We will restrict consideration to studies with experimental, quasi-experimental or longitudinal designs. Thus, we do not consider cross-sectional studies since it is difficult to separate out the casual role of microcredit in promoting health services because of various selection and reverse causation biases common to statistical evaluations of such studies as mentioned above. Even if microcredit is associated with positive health outcomes, we must ask whether the impact is greater than what would have been seen without microcredit.

The first type of studies includes those which simply measure health behaviors or status as outcomes after a microcredit intervention. The second includes those which have a health intervention in addition to microcredit. The latter has been the policy of the two large MFIs in Bangladesh–Grameen Bank and the Bangladesh Rural Advancement Committee. The rationale for this policy by these MFIs is that when women gather in their borrowing group, it is an ideal occasion for health education. Another option in microcredit provision that has been tried is to offer health services or health insurance at reduced cost for microcredit members.

The findings from these studies are also mixed. These findings are reviewed below.

A quasi-experimental study in Honduras and Ecuador compared behaviors of women who participated in conventional village banks with those of women who participated in a health bank. In the latter, women in bi-weekly meetings heard 15-min lectures on health topics (e.g., maternal health, management of illnesses, nutrition, immunization, etc.). Women who participated in the health banks had significantly less switching from breastfeeding to bottle feeding, but other health indicators were not significantly different between the two groups [29].

In the Dominican Republic, a longitudinal study had 3 non-randomized arms: (1) microcredit only; (2) health promotion only (e.g., management of acute respiratory infection and diarrhea, immunization, and breast and cervical cancer screening); (3) microcredit and health promotion [10]. Eleven health indicators were measured at baseline and a follow-up two years later. The health intervention included monthly visits to each family by trained health promoters. Eight of the eleven indicators significantly improved by the follow-up among those in the microcredit and health area while five of eleven improved among those in the health area only, and none improved among those with only microcredit.

A cluster randomized trial in South Africa included three study arms: (1) four villages with microcredit and an intervention on AIDS and gender equity; (2) four villages with microcredit only, and (3) four control villages. Of the three health-related indicators, none was significant in a comparison of microcredit only vs. control but two of the three HIV-related risk behaviors were significantly lower in the microcredit and health vs. control comparison [16].

An experimental study in one area of Peru had about half of loan groups randomized to receive eight 30-min sessions of health education. At the follow-up survey one year later, those in the intervention groups had significantly better health knowledge, but none of the eight child health indicators (e.g., anthropometry, reported diarrhea or respiratory infection) was significantly different between treatment and control groups [13].

In 138 villages in Benin an RCT with four randomized intervention groups was undertaken: (1) microcredit and health education of women; (2) microcredit with health education of mixed sex groups; (3) microcredit only for women; (4) microcredit for both women and men. Of six calculated health indices that were compared at follow-up three years later, no significant differences were seen between groups except on one HIV behavior score. The authors suggest that bundling health education with microcredit was not sufficient to improve health behaviors [15].

In a recent RCT in India, mandatory health insurance was introduced for individuals enrolled in an MFI in 101 villages that were randomly selected with another 100 villages serving as control. The health insurance intervention had no significant impact on health status, health care usage, or financing of health care expenditures, but this was largely because many persons in the intervention villages discovered that the health insurance was “mostly useless” and decided to drop their microcredit to avoid the health insurance imposition [6].

Several studies have examined contraceptive use as the outcome. An RCT in Ethiopia had four cells with villages randomly assigned to each cell: (1) microcredit only; (2) family planning only; (3) microcredit and family planning; (4) neither. This study found no effect on contraceptive uptake [32]. Among non-randomized studies, even when authors used sophisticated techniques, results have been contradictory. Thus, Pitt et al. [26] with national data from 87 villages in Bangladesh found no effect of microcredit participation on contraceptive use. But Steele et al. [31] with a before-after sample from one area of the country did find a program effect on contraceptive use. Both studies used econometric methods but the samples were different, possibly explaining the contradictory results.

Thus, the effects of microcredit or microcredit and health interventions combined on the health of individuals and families have been found to be positive in some studies and settings, but null in other studies and settings. The objective of the present study was to determine if the introduction of enhanced microcredit and basic health services separately and jointly in a randomized design would have any effects on health behaviors of individuals and families in rural Bangladesh.

## Methods

Our original study design was to introduce the program interventions in a random set of villages across Bangladesh that had neither microcredit nor non-governmental (NGO) health services. However, by the time of the start of the project in 2005, there were almost no villages in Bangladesh without microcredit. Indeed, in the 2004 Bangladesh Demographic and Health Survey (BDHS) [24], only 7 out of the 359 clusters visited, with an average sample size of 32 women interviewed in each, had no woman reporting participation in microcredit. Thus, we revised the design to have additional microcredit as an intervention. The logic was that if microcredit has positive effects, then provision of microcredit services to more households would show increased effects at the village level. Cluster randomization at the village level was the only feasible design. Grameen Bank was chosen as the partner to implement the interventions as it is well known for its microcredit work and had recently introduced health centers in some parts of the country to provide basic health services [1].

The 4-celled design of the study is shown in Table 1. To determine the needed sample size, the criterion variable chosen was contraceptive use. A baseline level of 50% was assumed (in the 2004 BDHS, the prevalence of contraceptive use among rural women was 56.7%, and modern contraceptive use was 46.0%). We wished to detect a change of 15% with Type I and Type II error probabilities of 0.05 and 0.20, respectively. From the BDHS with rural clusters (villages), we estimated the intra-cluster correlation to be 0.052. With these estimates and utilization of the software of Hayes and Bennett [14], we calculated that a design with 128 villages and at least 29 women interviewed per village would provide an adequate sample size for the study—approximately 930 women per study arm (see Table 1).

**Table 1:** Study design: Minimum sample size—number of villages (women) for before and after surveys for the four intervention arms

Additional microcredit worker	Improved health services	
	Yes	No
Yes	32 (930)	32 (930)
No	32 (930)	32 (930)

**Fig. 1:**
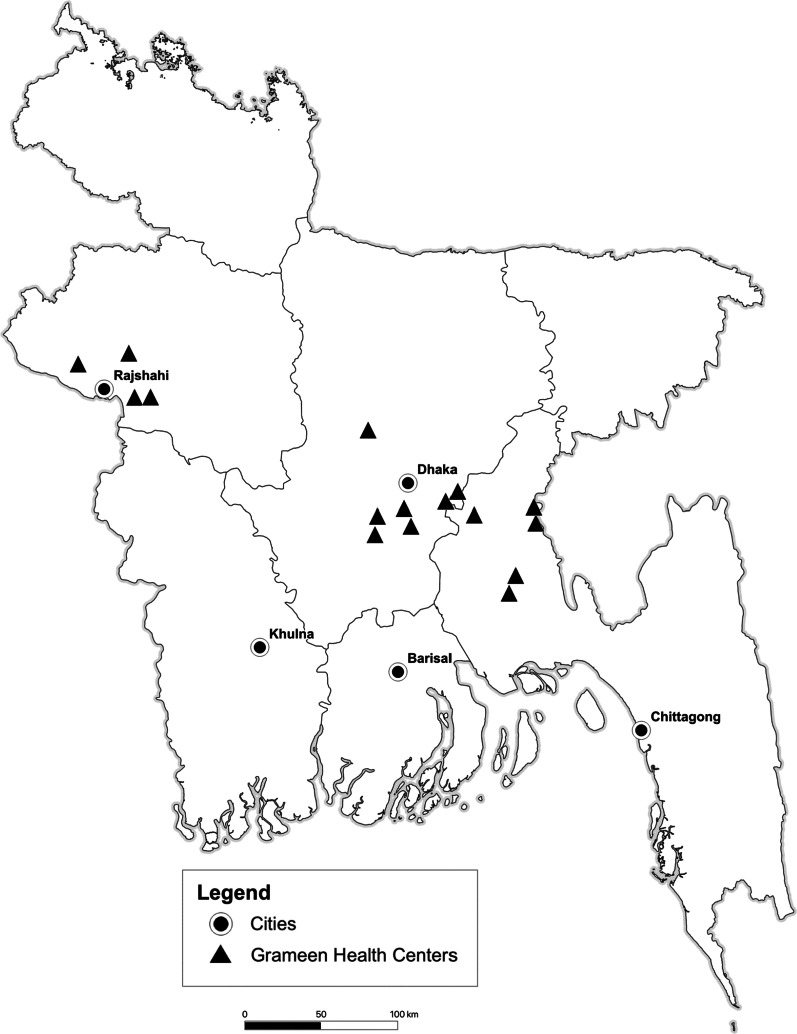
Map of Bangladesh showing location of Grameen Kalyan Health Centers

**Fig. 2:**
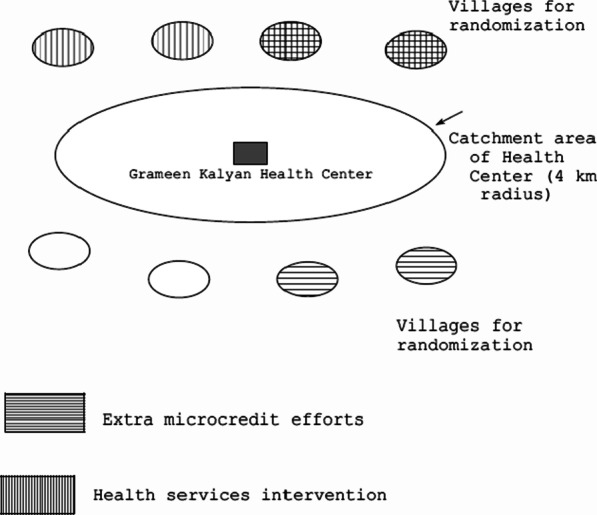
Schematic diagram showing Grameen Kalyan Health Center, its catchment area and four villages outside the catchment area on two opposite sides, which were randomized to receive health services intervention and/or enhanced microcredit services or neither intervention (16 such areas in the design)

To utilize health facilities and staff available in Grameen health centers, we chose villages in the vicinity of such health centers. Of the 31 Grameen health centers operating in the country at the time (2005), we selected 16 with the lowest reported coverage of microcredit in the upazila (administrative area below the district in Bangladesh (like a county or borough in Western countries)) according to nationally available data [25]. Figure 1 shows the location of the 16 centers. Next, an enumeration of 24 villages in the vicinity outside the catchment areas of these health centers was done to find villages estimated to have less than 50% of households participating in microcredit and with only governmental health programs. The catchment area of a Grameen health center is approximately a circular area around the center with a radius of about 4 km. In this enumeration, elders in each village were asked if there was any NGO health program in the village and approximately what percentage of households participated in microcredit. These data along with GPS coordinates were utilized to select eight villages, with two sets of four outside and on opposite sides of the health center catchment area. This setup is shown schematically in Fig. 2.

After this selection, a census of households in each village was undertaken. From the census, households with ever married women were identified. In these households, we asked: number of persons in the household, amount of land owned, and whether any woman of the household ever belonged to a microcredit group. From these data, households in the villages were categorized into 3 strata: (1) not eligible for microcredit; (2) eligible and had accessed microcredit; (3) eligible but had not accessed microcredit. An eligible household was one that owned less than l/2 acre of land.

Randomization of villages to the four study areas was done by the team of researchers independently of Grameen Bank and of the baseline survey; it was done by the researchers at Johns Hopkins University using a list of random numbers. For each center, we first randomly selected one of the sets of four villages to receive the health services intervention. Then, among each of the two sets of four villages on opposite sides of the health center, two were randomly assigned to have the additional microcredit intervention.

A baseline survey was conducted among a stratified random sample of households in each of the 128 villages. In each village, interviews were attempted in 31 randomly selected households with the following distribution by strata defined from the census as noted above: 4 in stratum 1; 12 in stratum 2; and 15 in stratum 3. (In a few villages, one or two more than 31 interviews were completed; interviewers in the teams were assigned more than 31 interviews on the assumption that some households would be non-response.)

The household questionnaire included the following items: a listing of household members with their relationship to the head, sex, age and marital status; information on source of drinking water, type of toilet facility, possession of items (electricity, wardrobe, table, chair, watch, bed, radio, television, bicycle, motorcycle, sewing machine and telephone or mobile phone), number of rooms, materials used in wall and floor construction, type of fuel for cooking, amount of land owned, any food deficit during the previous year, amount of rice stocked after the last harvest and ownership of domestic animals (cows, goats, chickens, ducks, fish). One ever married woman in each household was then selected and consent obtained for interview. The woman’s questionnaire was modeled after the BDHS and included questions on the following topics: respondent’s background (e.g., age, schooling, religion), a pregnancy history, details about maternal and child health care, contraceptive use, recent childhood illnesses, details on participation in microcredit, decision-making in the household, a 3-year month by month reproductive calendar, fertility preferences, and domestic violence.

Fieldwork was carried out between July and September 2006. Both the census and survey were conducted by a professional survey agency. Since the baseline was done before the interventions began, the interviewers were obviously blind to the random assignment of any village. Thirty interviewers and supervisors (social science graduates who were experienced in survey methods) were recruited. They received training on the content of the questionnaires and techniques to establish rapport with the respondents but still maintaining the neutrality needed to obtain the most accurate data possible. All questionnaire data were entered using CSPRO [33] with range and consistency checks, as well as double entry for complete checking.

After the baseline survey, the interventions by Grameen Bank and its health section, Grameen Kalyan, began. Specifically, for the villages with the health services intervention, health assistants were hired and trained by Grameen Kaylan to deliver basic education and health services. Each female health assistant covered two villages and visited house to house. She provided (a) ORS packets, (b) vitamins, iron, paracetamol, deworming tablets, and/or metronidazole tablets, (c) basic health and nutrition education, (d) referrals for illnesses to the Grameen Kalyan Health Center, and (e) announcement of a satellite clinic held each month in the village. The satellite clinic was held in a location in the village that was provided to Grameen Kalyan at minimal charge. A doctor or paramedic from the Health Center provided free services to anyone from the village who presented for care during the 2–5 h s/he was there. In cases in which further care was needed, the patient was referred to the Grameen Kalyan Health Center or, for advanced care, to the closest government hospital.

For the additional microcredit intervention, the Grameen Bank area offices that covered the villages in question were sent a letter from the Grameen Bank Head office in Dhaka asking them to assign an additional worker to each of the selected villages. Villages on opposite sides of the same Grameen Kalyan Health Center were often covered by different area offices.

A project office was established in Dhaka with two persons who monitored the health interventions on a quarterly basis, visiting 4 of the 16 health centers and surrounding study villages each month. In addition to meeting with the health assistants and health center staff, some process data were collected. Specifically, information was collected on the satellite clinics (e.g., attending health person, duration of the clinic, number of patients seen, and medicines dispensed) and work of the health assistants (number of households visited, number of ORS packets sold, vitamins, paracetamol, metronidazole, iron and deworming tablets given). Monitoring of the additional microcredit effort was deemed unnecessary since Grameen staff are well versed in procedures for motivating and giving loans.

After approximately 3 years of the intervention, a follow-up survey was done in 2009. We decided to re-interview, to the extent possible, the women who had been in the baseline survey to provide longitudinal data at the individual level. For the women who had changed residence, a tracking system was established to attempt to locate them in order to complete an interview. Specifically, from other family members still in the village or from neighbors, we asked for contact information of the woman—this was typically a cellphone number. In addition, to account for inevitable loss to follow-up, two “replacement” interviews were attempted in each village, selecting one household from each of strata 2 and 3, using the original (2006) household census data.

The questionnaire for the follow-up survey was mostly the same as that of the baseline, but with a few deletions and additions; the main addition involved details of women’s microcredit participation. The same survey organization carried out the follow-up survey and they were independent of Grameen Bank and interviewers were blind to the assignment of villages in the intervention. For the work, 36 interviewers were trained and 24 were selected. A “tracking” team was organized and charged with the task of locating women who had changed residence since 2006. A staff person from Johns Hopkins University monitored the quality of data collected during the first 3 weeks of fieldwork. Data were entered in CSPRO and transferred to STATA for analyses. Baseline and follow-up data were matched for the same household and woman.

Census data for each village were used to construct weights for each household and woman in the baseline survey. For the follow-up survey, response rates varied by strata, so for the analyses of women in both surveys, the baseline weight was adjusted by the factor l/r_j_, where r_j_ is the follow-up completion rate in strata j. Weighted analyses and adjustments for cluster effects were done using SVY commands in Stata [30].

To test whether the random allocation of village groups resulted in balance of characteristics between study arms, we compared village characteristics (a market in the village; a clinic within 2 miles; a hospital within 5 miles), household characteristics (wealth, food deficit during the year; electricity, own land, improved water source and whether own a cow and/or goat) and woman characteristics (work for pay, antenatal care for last birth, want no more births, ever attended school, age, and number of pregnancies). Then, to test for selectivity of women with completed baseline and follow-up interviews, we compared characteristics of these women with those of women interviewed in the baseline survey only. The characteristics examined were: age, level of schooling, parity, household asset score and marital status.

To construct the wealth index, binary asset indicators were chosen. These were: the presence or absence of electricity, a wardrobe, table, chair, clock, bed, radio, television, bicycle, at least one of a motorcycle, sewing machine or telephone, brick, cement or tin walls, and modern toilet or pit latrine. In addition, the ratio of the number of people in the household to the number of rooms in the house was used. Principal components analysis was employed to combine the indicators into an asset index [11]. The first principal component, which accounted for 32% of the variance, was utilized. The analysis yielded a score for each household. These scores were ordered and used to divide households into quintiles**,** representing their relative wealth with respect to other households in the study. This asset or wealth index reflects disparities that are primarily economic [12].

In the original protocol, four individual-level outcomes for the experiment were specified: contraceptive use, full immunization, trained birth attendant and empowerment index. We wanted to stay close to these in the analyses. For full immunization, we chose measles vaccination since it often indicates full immunization and allows a larger age group and thus larger sample size than is the case with other vaccines. Contraceptive use and trained birth attendant outcomes were kept as is. Regarding women’s empowerment, unfortunately we were unable to test for changes because there were unforeseen data collection differences between the baseline and follow-up surveys. A food security variable was added as an outcome because we heard anecdotally during the study that conditions had improved in the country between 2006 and 2009 and we wanted to see to what extent this was the case, and food (rice) stocks is an excellent indicator of a household’s economic well-being. We also tabulated microcredit participation, i.e., one of the two interventions, to see how it varied among the study arms.

Details of the outcome variables are:

Food security. For all ever married women, this was coded 1 if the household in which she resided did not experience any food deficit in the previous year and 0 otherwise.

Microcredit participation. For all ever married women, this was coded 1 for those currently a member of a microcredit organization and 0 otherwise.

Contraceptive use. For all married women ages 15–49 (at both rounds), this was coded 1 for those using a modern method of contraception and 0 otherwise.

Skilled birth attendant. For women who had a delivery in the past 3 years, this was coded 1 if there was a trained health worker assisting (Doctors, nurses, midwives, paramedics, and health assistants were defined as skilled birth attendants) and 0 otherwise.

Measles immunization. For all women with children between the ages of 12 and 23 months of age, this was coded 1 if the child had received a measles immunization (with this information either from a vaccination card or from mother’s recall) or 0 otherwise.

Since only a small proportion of women were lost to follow-up (see below), we chose to analyze changes with data from women interviewed in both the baseline and follow-up surveys. When the sample is the same women at two time points, to test for changes we used McNemar’s test with unweighted data [21]. For the comparisons that involved different women at baseline and follow-up, we utilized a z-test of proportions. (The actual variance of the difference of proportions p1 and p2 is V(p1) + V(p2) − 2*Cov(p1,p2), but since the covariance in such situations is almost always positive, we believe that the decrease of variance here offsets the increase in variance due to the design effect.)

The study was approved by the Institutional Review Boards of the Johns Hopkins School of Public Health and the Bangladesh Medical Research Council. Informed consent was obtained before conducting an interview. (Since the questionnaire included a section on domestic violence, special arrangements were made to have a counselor available at the closest health center to whom women who reported recent violence could be referred by the interviewers.)

Analyses for RCTs are relatively straightforward. With cluster level randomization we used intent-to-treat analyses. First, we tabulate the outcome measures at baseline and follow-up surveys by study arm and test for differences. Since this was an RCT, the intervention groups were balanced on outcomes at the baseline except for one variable–women’s education (see below). Therefore, difference of differences analyses was not needed and analyses of outcomes at follow-up were sufficient. Indeed, difference of differences analyses in this case has been shown to have low power [22].

Since all outcomes were binary, we used logistic regression for each outcome from the follow-up survey with indicator variables for study arm. Odds ratio estimates are presented. Since there were significant differences in the proportion of women who had ever attended school by study arm, we include this as a covariate in the regressions. All these analyses used the SVY commands in Stata, which adjusts for both clustering and sample weights.

## Results

A comparison of baseline characteristics of villages, households and eligible women by intervention arm is given in Table 2. The only variable with significant differences across the study arms was whether the woman had ever attended school—the percentages were significantly higher in the health services and in the combined intervention village groups than in the other two arms. We therefore adjusted for this variable in the analyses.

**Table 2:** Village, household and eligible women characteristics (percentages in category) at baseline, by intervention assignment

Level and characteristic	Intervention group				
	All groups	Control, i.e., none	Microcredit only	Health services only	Both interventions
*Village level (n* *=* *64)*					
Market in village	50	38	50	53	59
Clinic within 2 miles	60	56	63	75	53
Hospital within 5 miles	44	34	41	50	53
*Household level (n* *=* *3998)*					
Poor (bottom three quintiles)	60	64	53	58	64
Not food deficit during the year	64	66	60	67	66
Electricity in the home	62	66	63	59	60
Own land	45	47	38	47	49
Improved water source	57	63	54	59	52
Own cow and/or goat	47	48	44	46	51
*Woman level (n* *=* *3933)*					
Currently works for pay	10	10	12	10	10
No ANC for last birth	18	15	19	15	21
Want no more births	42	44	45	43	39
Ever attended school *	54	47	45	60	60
Age (mean)	35.5	36.4	35.5	34.6	35.6
Number of pregnancies (mean)	4.0	4.2	4.1	3.9	4.1

*p < 0.05 for test of equality of proportions between study arms

**Fig. 3:**
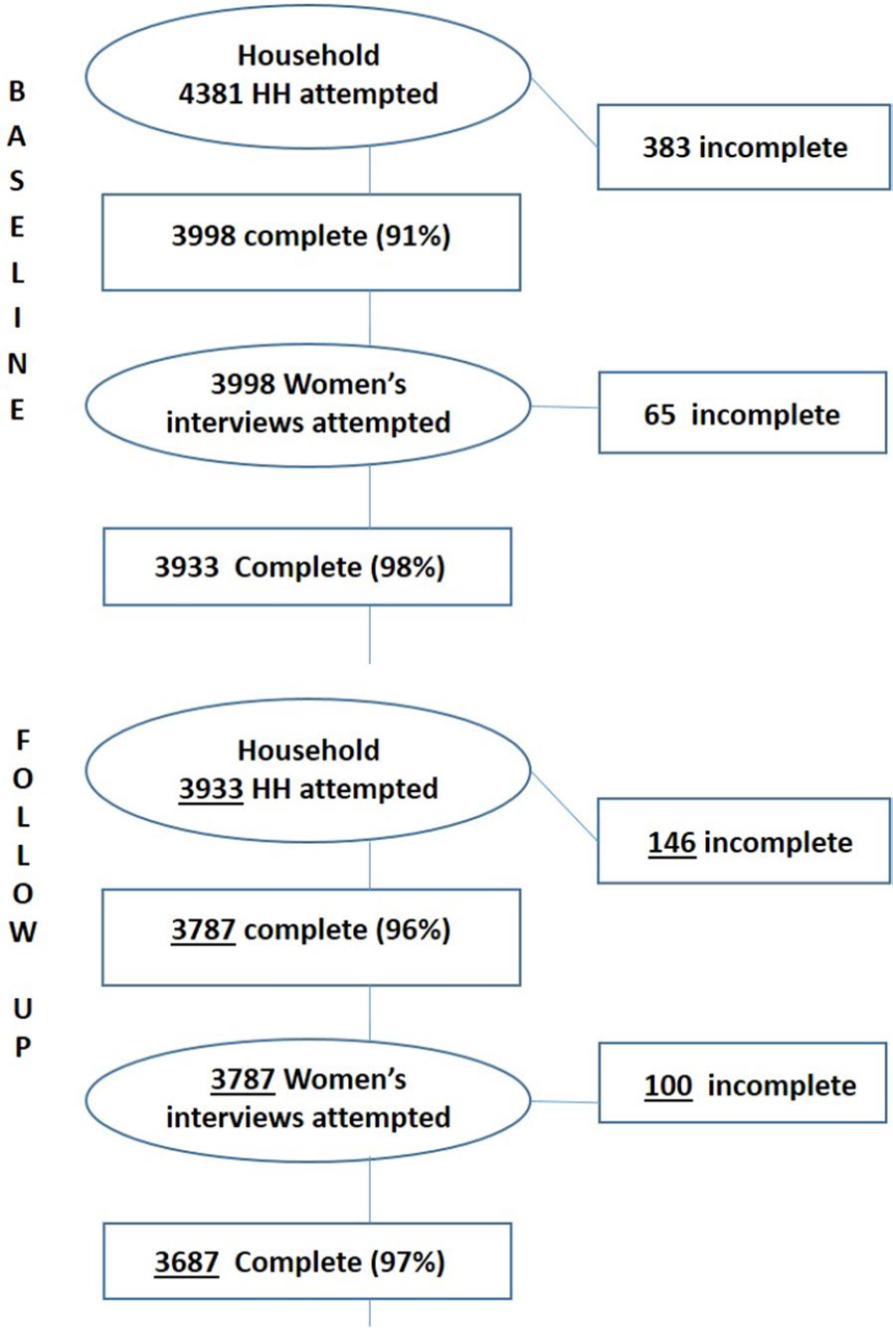
Flowchart showing attempted and completed household and women’s questionnaires in the baseline survey of 2006 and follow-up survey of 2009

Figure 3 is a flowchart of study participants in the baseline and follow-up surveys. Of the 4381 households sampled for the baseline survey, 3998 were completed (91.3% response rate) and in these households, 3933 women were interviewed (response rate of 98.7%). Of these, 3687 (93.7%) also had completed follow-up questionnaires. For those 246 women lost to follow-up, the reported reasons were: respondent migrated (54%), respondent incapacitated or died (13%), respondent not at home (15%) and other (17%). There was no significant difference in attrition by study arm (not shown). A comparison of characteristics of those lost to follow-up with those re-interviewed is given in Table 3. The women lost to follow-up were in households with significantly lower asset scores and were more likely to be widowed at baseline. A slightly greater percentage of those lost to follow-up had above primary schooling, but the difference was not statistically significant at the p = 0.05 level.

**Table 3:** Baseline survey characteristics of women by whether they had completed follow-up interviews or not

Baseline characteristic	Status of follow-up interview		*p* value (unweighted)
	Complete	Incomplete	
Number of women	3687	246	
*Means*			
Age (years)	34.2	35.0	0.49
Parity	3.7	3.5	0.37
Household asset score	0.01	− 0.29	< 0.01
*Percent distributions*			
Schooling: none	57.3	58.5	0.10
Primary	25.9	20.7	
Above primary	16.8	20.7	
Marital status: married	89.4	82.5	< 0.01
Widowed	8.9	13.0	
Other	1.7	4.5	

Baseline and follow-up values of the five outcome variables by study arm are given in Table 4. The results for each outcome are considered in turn.

1. The percentages of households with food security during the entire year before the survey increased significantly in all four study arms.
2. The level of microcredit participation did not change significantly in any of the study arms. There was actually a decline in microcredit participation in both arms with the additional microcredit intervention.
3. Contraceptive use increased significantly in the control villages, but changes in the intervention areas were not significant.
4. The percentage of women having a trained birth attendant at the last delivery increased significantly in the health-services only area and there were no significant changes in the other study arms.
5. For the percentage of children 12–23 months with measles immunization, there was no significant change in any study arm.

**Table 4:** Estimates of five outcome variables at baseline, follow-up and the difference and significance tests of change, by study arm

Study arm	Sample size	Value of indicator at		Difference (Fup-Bs)	McNemar’s *p* value
		Baseline	Follow-up		
*Percentage of households not food deficit during year (food security)*					
All areas	3687	67.4	85.6	18.2	0.001
Control*	937	67.5	87.1	19.6	0.001
MC-only*	921	72.0	85.5	13.5	0.001
HS-only*	914	62.8	83.1	20.3	0.001
MC and HS*	915	67.1	87.2	20.1	0.001
*Percentage currently participating in microcredit*					
All areas	3687	31.6	30.7	− 0.9	0.87
Control	937	31.3	33.2	1.9	0.90
MC-only	921	30.2	26.9	− 3.3	0.26
HS-only	914	35.1	35.8	0.7	0.74
MC and HS	915	29.6	27.8	− 1.8	0.19
*Percentage currently using contraception (currently married women age 15–49)*					
All areas	2839	64.6	67.6	3.0	0.17
Control*	728	64.8	69.6	4.8	0.02
MC-only	721	65.0	66.4	1.4	0.48
HS-only	705	65.3	68.1	2.8	0.15
MC and HS	685	63.4	66.3	2.9	0.17

Study arm	Sample size (baseline, follow-up)	Value of indicator at		Difference	*p* value
		Baseline	Follow-up		
*Percent with trained birth attendant at last birth*					
All areas	1164, 915	31.6	36.4	4.8	0.06
Control	302,235	33.2	28.5	− 4.7	0.37
MC-only	301,243	31.4	33.0	1.6	0.18
HS-only*	283,223	29.1	43.9	14.8	< 0.01
MC & HS	278,214	33.5	39.7	6.2	0.32
*Percent of children 12–23 months old with measles immunization*					
All areas	377,268	87.9	86.6	− 1.3	0.33
Control	95 61	88.5	93.5	5.0	0.14
MC-only	90,70	84.6	79.9	− 4.7	0.28
HS-only	92,69	90.1	83.1	− 7.0	0.19
MC & HS	100,68	90.0	88.7	− 2.3	0.46

*p < 0.05 for test of the hypothesis of no change between baseline and follow-up surveys

The finding that microcredit participation actually declined in the microcredit and microcredit plus health services arms was completely unexpected. As an explanation for this finding, there was a non-trivial problem that occurred in implementing the microcredit intervention. The study design called for selection of eight villages outside the catchment area of each Grameen Health center and, in the randomization, half of these would receive enhanced microcredit, specified as an additional microcredit worker assigned to those villages. As noted above, at the beginning of the study, the Grameen Bank headquarters in Dhaka sent a letter to each concerned Grameen Bank office regarding implementation with an additional worker. While the design was good, it was problematic from the perspective of implementation by Grameen Bank. The four villages randomized to receive additional microcredit around a Health Center were often covered by two or even three separate Grameen area offices; this led to confusion and the coordination of where an extension worker was assigned was haphazard. Unfortunately, this confusion led to decreased microcredit activity in the microcredit intervention villages. Since funding for this microcredit extension worker was a Grameen Bank contribution to the project, there was no control on its implementation by the project staff. Given these results, for the multiple logistic regressions, we collapsed the intervention groups to two: control including the original control and microcredit only arms and health services including the health services only and health services plus microcredit arms.

Table 5 gives the regression results for the four key outcome indicators at the follow-up survey. The covariate for whether the woman had ever attended school had significant positive effects for both food security and measles vaccination. After adjustment for women’s schooling, there were no significant effects of the health services intervention on any of the outcomes. Results were similar when the regressions were done without the schooling covariate and also when the four intervention arms were used instead of two (neither is shown but both are available upon request).

**Table 5:** Estimated odds ratios (and 95% confidence intervals) from logistic regression fit of four outcome variables on experimental and control areas

Covariate	Outcome			
	Food security (n = 3687)	Contraceptive use (n = 2960)	Measles vaccination (n = 268)	Trained birth attendant (n = 915)
Control areas^a^	1.00	1.00	1.00	1.00
Health services areas^b^	0.79 (0.51, 1.24)	0.79 (0.48, 1.31)	0.60 (0.14, 2.59)	1.18 (0.58, 2.44)
Woman’s schooling (ref. = none)	2.51** (1.66, 4.21)	0.48 (0.58, 1.23)	5.14** (1.66, 14.27)	1.38 (0.70, 2.71)
Constant	4.38 (3.24, 5.91)	1.96 (1.34, 2.86)	3.00 (1.09, 8.29)	0.41 (0.20, 0.83)

**p < 0.05 for test that odds ratio = 1.0

^a^Including the original control villages and the microcredit-only villages

^b^Including the original health services villages and the health services plus microcredit villages

## Discussion

In this study, only one of the three health behaviors showed significant improvement over time in the health services intervention villages but not in the control or other arms. The significant result was for presence of a trained birth attendant at last delivery. This result could attest to the effectiveness of the health education imparted by the health assistants about the importance of having trained personnel at birth and/or to the villagers availing themselves of the health facility or health personnel of the Grameen Kalyan Health Centers which they had learned about from the health assistant and/or the medical person during the satellite clinics. However, in the logistic regressions of the follow-up data, the effect of the health services intervention was not significant for any of the three health behaviors. One reason for the finding of significant differences between levels of the outcomes over time but no significant effects in the logistic regressions is that in the former, the design effect did not contribute since it was the same villages at both time points, but in the logistic regression it did and design effects were quite large (the minimum value was 3.8 for measles immunization and maximum was 9.8 for contraceptive use).

For the health interventions generally, the project provided the funds for the extra health worker in the randomly selected villages and this was closely monitored. However, because the project health assistants were based in the Grameen Kalyan health center, which was a considerable distance from the experimental villages, travel to the study villages often took several hours each way. Furthermore, village health workers were at the time paid 3000 taka (46 USD) per month, which after funds for a pension and travel were subtracted, became about 2000 taka (31 USD). The salary was in line with pay of health assistants already attached to the Grameen Health Centers, but the latter worked in villages within the health center catchment area, i.e., they had much shorter travel times. The Grameen Kalyan workers also had some level of job security within the Grameen system which the temporary health assistant staff of this project did not have. Thus, there was considerable turnover in health assistants of the project and their morale was low. Almost 3/4 of the way through the project when this problem became clear, their salaries were raised to 4000 taka which helped with retention.

The nonsignificant results of this study are in line with results from other RCTs of microcredit interventions in diverse settings reviewed above though only the study in Ethiopia included a health (family planning) arm and an arm with both interventions. But there too, of the 40 outcome variables considered, only five showed significant effects of the interventions [32].

The RCT is taken as the “gold standard” in medical research and the same has generally been assumed in social science research. However, several points in critiques of RCTs involving human behavior change deserve consideration [20, 28]. Most importantly, results from an RCT typically cannot provide much insight on the “how and why” of any significant effects that are found. Human behavior change involves complex pathways between intervention and outcome. Combining qualitative methods with an RCT is one proposal to try to understand the how and why of the findings. Of course in the present case the finding of no effects of the interventions is perhaps simpler to explain due to the problem of implementation of the microcredit intervention described above.

Several weakness of the study deserves mention. First, there was variability or heterogeneity in implementation. In particular, for the microcredit intervention, though the overall effect on microcredit participation was negative, villages assigned to additional microcredit around some Health Centers did show positive effects. Also, some health assistants had greater motivation and enthusiasm for their work than others and this could have affected what they were able to convey to women in the study villages (see “Appendix”). In short, the interventions were not uniformly applied; but this is to be expected with fairly large-scale social science interventions. Second, sample size was reasonable but not large enough to detect small differences. For example, there were only 268 children between the ages of 12 and 23 months at follow-up–the denominator for the outcome of measles immunization. It might also have been useful to compare treatment of diarrhea across the study arms (since ORS packets were made available by the health assistants in intervention villages), but only 47 mothers reported diarrhea in a child in the two weeks before the baseline survey.

The study had a number of strengths. First, though the sample was not representative of rural Bangladesh because villages had to be in the vicinity of a Grameen Health Center where the health assistants were available and the medical personnel for the satellite clinics were based, the 128 villages were from three of the seven divisions of the country. Second, the project staff were able to monitor the work of the health assistants throughout the project and were able to identify and address problems when they arose. Third, the same survey organization carried out both the baseline and follow-up surveys and response rates were quite high in both surveys (see Fig. 3). Fourth, in the follow-up survey we attempted to re-interview all women interviewed in the baseline and, with tracking efforts, were able to reach 94% of the original sample. Finally during a one-day seminar, the results of this study were shared with Grameen Health and Grameen Microcredit officials as well as other stakeholders of health and Microcredit agencies in Bangladesh including the Director General of the Palli Karma Sahayak Foundation, the government’s lead microcredit agency, so they could learn from this experiment.

## Conclusion

The results of this study are in line with the results of other RCTs of microcredit or microcredit combined with health interventions with largely negative results, (i.e., few null hypotheses could be rejected). Implementation of the additional microcredit intervention was poor but the study arm with health workers visiting households door-to-door did produce positive results on the likelihood of delivery assisted by a trained birth attendant. Close monitoring of interventions is essential in such experimental programs so that researchers can determine if the intervention adheres to what is outlined for the study and it is possible to properly associate cause with effect at the time of analyses.

While RCTs minimize biases of self-selection and non-random program placement associated with many other study designs, they cannot provide answers to why a given change occurred, so combining with qualitative research is necessary. Of course, it is impossible to know beforehand which behavior will change significantly; therefore, probably the most useful qualitative research would be done afterward. For example, in the present case, qualitative interviews could be with a select group of women who had delivered with a trained attendant as well as with a select group of women who did not, as documented in the follow-up survey. The RCT can provide an estimate of the magnitude of the change in behavior and the qualitative research can give insights on how and why the change occurred.

## Data Availability

The original data and complete codebook are available from SB. There are plans to have it archived with ISPCR.

## Appendix

After about twenty months of implementation of the project, it became apparent from the regular monitoring by the JHU project staff, that the work of the health assistants was not meeting the standard set for the design. Since they had to travel long distances to reach the villages, they did not have adequate time to talk with village women individually and some lacked adequate knowledge of treatment for common illnesses. After extensive consultation with local and outside experts, it was decided that a special training of health assistants was warranted. In conjunction with the Grameen Bank and the International Centre for Diarrheal Disease Research, Bangladesh, a 3-day training in the Integrated Management of Childhood Illnesses (IMCI) was given. To adequately allow estimation of the effect of this further training, we decided to train health assistants from 8 of the 16 centers, which were randomly selected. We distinguished the two interventions in some analyses. However, there were no significant differences, so those results are not presented here. This might have been expected since only ten months remained in the project when the training was done and sample sizes were insufficient to detect anything other than quite large differences. Also, with a fixed budget we were unable to increase the number of workers to compensate for the long travel time it took to reach villages so workers would have more time to talk with the village women.

## Abbreviations

DHS: Demographic and Health Survey
MFI: Microfinance Institution
RCT: Randomized controlled trial
